# Exploring the Impact of Intermolecular Interactions on the Glassy Phase Formation of Twist-Bend Liquid Crystal Dimers: Insights from Dielectric Studies

**DOI:** 10.3390/molecules28217441

**Published:** 2023-11-06

**Authors:** Antoni Kocot, Małgorzata Czarnecka, Yuki Arakawa, Katarzyna Merkel

**Affiliations:** 1 Institute of Materials Engineering, Faculty of Science and Technology, University of Silesia, 75 Pułku Piechoty 1a, 41-500 Chorzów, Poland; antoni.kocot@us.edu.pl; 2 Faculty of Electrical Enginesering, Automatics, Computer Science and Biomedical Engineering, AGH University of Science and Technology, 30-059 Krakow, Poland; malgorzata.czarne@gmail.com; 3 Department of Applied Chemistry and Life Science, Graduate School of Engineering, Toyohashi University of Technology, Toyohashi 441-8580, Japan; arakawa@tut.jp

**Keywords:** liquid crystal dimers, dielectric spectroscopy, glassy phase formation

## Abstract

The formation of the nematic to twist-bend nematic (N_TB_) phase has emerged as a fascinating phenomenon in the field of supramolecular chemistry, based on complex intermolecular interactions. Through a careful analysis of molecular structures and dynamics, we elucidate how these intermolecular interactions drive the complex twist-bend modulation observed in the N_TB_. The study employs broadband dielectric spectroscopy spanning frequencies from 10 to 2 × 10^9^ Hz to investigate the molecular orientational dynamics within the glass-forming thioether-linked cyanobiphenyl liquid crystal dimers, namely, CBSC7SCB and CBSC7OCB. The experimental findings align with theoretical expectations, revealing the presence of two distinct relaxation processes contributing to the dielectric permittivity of these dimers. The low-frequency relaxation mode is attributed to an “end-over-end rotation” of the dipolar groups parallel to the director. The high-frequency relaxation mode is associated with precessional motions of the dipolar groups about the director. Various models are employed to describe the temperature-dependent behavior of the relaxation times for both modes. Particularly, the critical-like description via the dynamic scaling model seems to give not only quite good numerical fittings, but also provides a consistent physical picture of the orientational dynamics in accordance with findings from infrared (IR) spectroscopy. Here, as the longitudinal correlations of dipoles intensify, the **m**_1_ mode experiences a sudden upsurge in enthalpy, while the **m**_2_ mode undergoes continuous changes, displaying critical mode coupling behavior. Interestingly, both types of molecular motion exhibit a strong cooperative interplay within the lower temperature range of the N_TB_ phase, evolving in tandem as the material’s temperature approaches the glass transition point. Consequently, both molecular motions converge to determine the glassy dynamics, characterized by a shared glass transition temperature, *T_g_*.

## 1. Introduction

The simplest liquid crystalline mesophase, the uniaxial nematic (N) phase, is characterized by the orientation of the molecules in a preferred direction, but with low positional order. The introduction of chirality, either through a chiral center in the mesogen or by adding a chiral dopant to the N phase of the host, results in the formation of a chiral nematic (N*) phase that has a helical structure. Independently, Meyer [1] and Dozov [2] predicted that liquid crystal phases with local chirality could be formed with bent achiral mesogens. The helical phase could occur spontaneously as a result of the simultaneous bending and twisting of a local director in an array of non-chiral molecules. The resulting twist-bend nematic (N_TB_) phase is present in an equal number of degenerate domains with opposite twist directions and the director is tilted relative to the helical axis. This new nematic-type phase is distinguished by unusual periodic patterns observed with polarizing microscopy and very fast electro-optic switching in the microsecond regime [3,4]. However, an unambiguous determination of the phase structure appears to be very difficult, since it exhibits no electron density modulation. Extensive studies [5,6,7] suggested that this structure corresponds to a phase with a spontaneous conical twist-bend director distortion, which was theoretically predicted to be driven by an elastic instability with the sign inversion of the bend elastic constant K_33_ for bent-shaped molecules [2].The N_TB_ phase was first discovered for 1,7-bis-4-(4′-cyanobiphenyl)heptane, CB7CB, which consists of two mesogenic units connected with a flexible spacer [5,6,7,8]. Other dimeric mesogen [9,10,11,12,13,14,15,16,17] bent-core species [18,19,20] have been reported to manifest the N_TB_ phase. Despite the significant number of materials investigated, a general structure–property relationship for the N_TB_ phase remains elusive; however, it is found that a spatially uniform curvature of the molecule is necessary to form the N_TB_ phase regardless of the underlying chemical groups [21,22].

The primary motivation for this work is the analysis of the orientational dynamics of molecules in the nematic phases: N and N_TB_ formed with the polar symmetric dimers CBSC7SCB and the similar asymmetric dimers CBSC7OCB. Dielectric relaxation measurements were carried out for both samples in the entire temperature range up to the glass transition. The observed relaxation processes, as in earlier dielectric relaxation studies [5,20,23,24], were interpreted using different molecular motions defined with theoretical models of dielectric relaxation [25,26,27]. In both nematic phases, the rotation of dipolar groups associated with the terminal cyanobiphenyl groups lead to two relaxation modes related to the rotational dynamics of the molecules. The low-frequency mode, denoted **m**_1_, is caused by “end-to-end” motion of dipolar groups, which are parallel to the mesogen axis. The high-frequency mode, denoted **m**_2_, is the result of the precessional rotation of the dipolar groups around the director. These modes contribute to the complex dielectric permittivity differently depending on the orientation of the dielectric relative to the measured electric field. 

The important part of the analysis concerns the study of the glass transition identified in both longer dimers clearly below the N-N_TB_ transition. The glass transition can be characterized by the temperature *T_g_* and the rate at which various properties of the material change with temperature as the liquid phase approaches the glass transition. For systems exhibiting a certain number of structural relaxations, it is possible to identify dielectric glass transitions corresponding to each of the two types of molecular motion independently. It is interesting whether changes in molecular interactions observed in previous spectroscopic studies [28,29] can be related to the observed glass transitions. The results for the CBSC7SCB and CBSC7OCB dimers presented here are discussed for information on which molecular movements are frozen during glass formation. The results for the symmetric and asymmetric liquid crystal dimers presented here are discussed to provide information on how the changes in molecular interactions observed near the N-N_TB_ transition and below affect the aggregation process and how they are frozen during glass formation. 

## 2. Results 

### 2.1. Dielectric Relaxation 

The simplest dielectric absorption model applicable to most materials is the Debye model, which describes the single dipole relaxation process. The temperature dependence of the relaxation time can be represented by the phenomenological Arrhenius equation, which introduces the activation energy *E*_a_ for the reorientation of a molecular dipole in a dielectric environment.

(1) $$\tau =\tau_{0}\;\exp[\;E_{a}/k_{B}T],$$

Although the Arrhenius equation describes the dielectric relaxation of many simple fluids well, there are several materials for which the equation fails and it is necessary to modify the equations in describing experimental results. One of the phenomenological equations most commonly used to describe the temperature dependence of relaxation time data (*τ*) is the Vogel–Fulcher–Tammann (VFT) equation [30]:

(2) $$\tau =\tau_{0}\;\exp[\;B/\left(T-T_{0}\right)],$$

Despite some concerns about the validity of the VFT equation, as it assumes dynamic divergence of the relaxation time at some finite temperature *T*_0_, some theories are based on the VFT equation such as the Adam–Gibbs entropy model (AG) [31] and some more recent theoretical models [32,33,34]. The basic idea behind these theories is that glass formation is associated with highly cooperative movements in a structurally fluctuating sample, with cooperativity growing as *T_g_* is approached. Combining the above ideas of glass formation with a mean-field description of the virtual phase transition, the dynamic scaling (DS) model [35,36] has been proposed such that

(3) $$\tau =\tau_{0}\;[\left(T-T_{c}\right)/T_{c}{]}^{-\Phi },$$

where the pre-factor *τ*_0_ is defined as the relaxation time at 2*T_c_*; the temperature *T*_c_ is the temperature of the virtual phase transition (the critical temperature), usually located slightly below *T_g_*. For the high-temperature dynamic domain, well above the glass transition where the coupling mechanisms can be disregarded, Mode Coupling Theory (MCT) provides a power law function similar to the above eq. but with fitting parameters of the different physical meanings to those reported by the DS model. In that case, *T_c_* accounts for the crossover temperature from the ergodic to the non-ergodic domain. The critical temperature *T_c_* seems to correlate with the caging temperature *T*_A_ [37].

An alternative non-divergence description that has recently been considered is the Waterton–Mauro (WM) parameterization, derived empirically by Waterton [38] in the 1930s and recently derived theoretically by Mauro and colleagues [39]; it is now considered a promising function for representing relaxation times, defined as follows:

(4) $$\tau =\tau_{0}\;\exp[K/T\;\exp\left(C/T\right)],$$

where *τ*_0_ has the same meaning as in the VFT equation; *K* and *C* parameters are related with effective activation barriers, being both defined as thermal activation fitting parameters.

The problem is more complex in dielectric studies of liquid crystals, because of their macroscopic anisotropy and the presence of a nematic potential. The solution of the rotational diffusion equation for a rigid dipolar molecule in the presence of a nematic potential predicts that the frequency dependence of each component of the electric permittivity can be characterized by two exponential decays [25,26]. Rotation of the molecule about a short molecular axis in the presence of the nematic potential gives rise to the low-frequency relaxation mode **m**_1_ detected in the parallel component of the permittivity, which is classified as *τ*_00_ in terms of the spherical harmonics. Relaxation of the longitudinal component of the molecular dipole with precession about the director axis contributes to a high-frequency mode, **m**_2_, detected in the perpendicular component of the permittivity and is classified as *τ*_10_ in terms of the spherical harmonics. This is for a nematic potential assumed to be of the form

(5) $$U/k_{B}T=\sigma \cos^{2}\theta ,$$

where *σ* is proportional to the uniaxial order parameter *S* (for *S* < 0.6)*,* while approximate expressions for the relaxation times can be derived [26] as

(6) $$\sigma =\frac{3}{2}S\left(5-\pi S\right)/\left(1-S^{2}\right),$$

The nematic potential barrier parameter, *σ*, is defined as *σ = q/*R*T* where *q* is the height of the barrier separating two minima along the ***n*** direction.

(7) $$\frac{\tau_{00}}{\tau_{D}}=\left(\frac{e^{\sigma }-1}{\sigma }\right){\left(\frac{2\sigma \sqrt{\delta /\pi }}{1+\sigma }+2^{-\sigma }\right)}^{-1},$$

(8) $$\frac{\tau_{10}}{\tau_{D}}=\frac{1-S}{1+S/2},$$

The relaxation time *τ***_D_** is that for rotational diffusion in the isotropic phase. Longitudinal, *μ*_l_, and transverse, *μ*_t_, components of the molecular dipole moment, *μ*, contribute to the dielectric permittivity differently and they relax at different frequencies of the probe field. In solving the rotational diffusion equation, a uniaxial nematic potential has been assumed. An inconsistency arises here, since molecules that have a dipole inclined to the long molecular axis are intrinsically biaxial.

### 2.2. Molecular Modes in the Nematic Phase

The dynamic dielectric responses of CBSCnSCB and CBSCnOCB (n = 5, 7) were measured in metal cells with a 50 μm spacer, in the frequency range of 5 Hz to 1 GHz [40]. The dielectric response of CBSCnSCB and CBSCnOCB in the nematic phase shows two relaxation processes, the contribution of which to the dielectric spectrum depends on the orientation of the director in the measurement cell. These dielectric relaxations can be related to the rotational diffusion of dimer molecules: the reorientation of end-to-end dipolar groups parallel to the director at low frequencies (**m**_1_), according to the theoretical model of dielectric relaxation in nematic dimers [25,26], and the precessional movement of dipolar groups around the director for the high-frequency branch of the spectrum (**m**_2_).

As already stated, the *ε*″ spectra at higher frequencies are dominated with two maxima. These correspond to the two molecular relaxation modes **m**_1_ and **m**_2_ of the symmetric CBSCnSCB and asymmetric CBSCnOCB dimers, as also made for CBCnCB by Cestari et al. [5], Merkel et al. [24], and López et al. [41]. We analyzed the two observed processes, in the N phase, and we were enabled to describe them unequivocally using an orientational order, *S*, and so-called Debye relaxation time, which corresponds to the relative relaxation in the absence of orientational order. This can be simply interpreted as an extension of the isotropic relaxation time into the temperature range of the nematic phase. Following Equation (4), the contribution to the perpendicular component of permittivity originates from the precession rotation of the transverse component of the dipole moment, i.e., rotation of the bow axis of the bent-core conformation contributes significantly to the perpendicular component.

This is the assignment of mode **m**_2_. The higher frequency relaxation process, **m**_2_, can arise from the rotations of a segment of the molecule, i.e., the internal rotation of each monomer with the spacer anchored involves the fluctuations of the cyanobiphenyl dipolar moment. As the length of the spacer in between the two mesogenes in the dimer is large enough, such an independent internal rotation of each monomer of the dimer is highly feasible. The temperature dependencies of *δε* and of the relaxation time $\tau_{10}$ are suggestive of the precessional rotation of the longitudinal component of each tio-cyanobiphenyl (SCB) dipole moment around the director in a planar-aligned cell.

**Figure 1:**
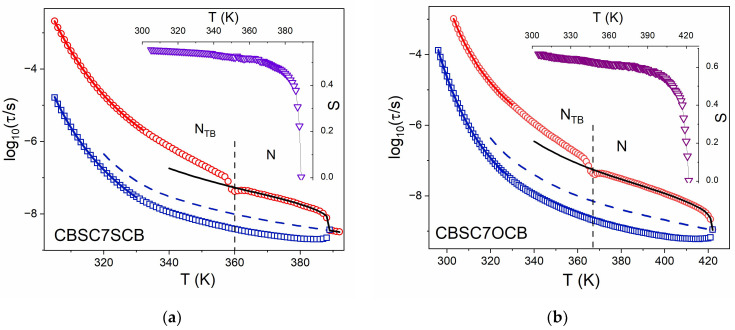
Plots of relaxation times for modes **m**_1_ and **m**_2_ (5 μm planar cell): (**a**) symmetric dimer (CBSC7SCB), (**b**) asymmetric dimer (CBSC7OCB). Symbols: red circles (**○**)—**m**_1_ mode, dark blue squares (**□**)—**m**_2_ mode, rotational diffusion model fitting: black solid line—**m**_1_ mode, dash blue line—*τ*_D_ relaxation time, solid blue line—VFT model [30], solid red line—fitting of experimental data by Equation (2), purple/burgundy triangles (**∇**, **∇**)—order parameter, S (as an insert).

Relaxation times of the modes, however, seem to be well defined solely with the local orientation order in the nematic phase. All dimer dynamics behavior in the nematic phase is quite well reproduced with the molecular dynamic model (Figure 1a,b). The experimental relaxation times, *τ*_00_ and *τ*_10_, for mode 1 and 2, respectively, were well fitted using Equations (6)–(8), assuming *S* and *τ***_D_** as unknown values. Results are shown in Figure 1a,b. Temperature dependences of the orientational parameters correspond quite well to the results obtained with infrared spectroscopy [29], but their values are slightly smaller than referenced because dielectric ones are related to the S-CB or (O-CB) dipole moments but IR ones correspond to the molecular axis.

In the N_TB_ phase, the temperature dependences of the *τ*_10_ of the **m**_2_ mode keep their trends without any step at the transition temperature, irrespective of if the director becomes tilted with respect to the symmetry axis. It seems this process is exclusively determined with the local orientational order (relative to the local director), which is continuously growing on decreasing temperature. This is not the case for end-over-end relaxation time, *τ*_00_, of the mode **m**_1_. First of all, relaxation time dependence clearly shows a kink at the transition temperature, which indicates an increased relaxation rate at the transition temperature. This is more likely due to critical fluctuations of the director in the vicinity of transition [42]. Then, in the N_TB_ phase, the temperature trend of the *τ*_00_ relaxation time suddenly increases, with respect to the trend in the nematic phase. We can simply interpret this fact as an apparent growth of the potential barrier, *q*, separating two minima along the ***n*** direction, upon entering the N_TB_ phase [26,38]. This finding corresponds well with an orientational correlation effect of the longitudinal dipoles ($g_{\left|\right|}\neq 1$), which was revealed with IR spectroscopy [28,29].

Models for the interpretation of the low-frequency relaxation in liquid crystals are often based on a single particle relaxation process, but spectroscopic probes of molecular motion such as magnetic resonance, neutron scattering, and time-resolved fluorescence depolarization suggest that reorientation times for mesogens are of the order of 10^−9^ s to 10^−10^ s in isotropic, nematic, and disordered smectic phases. Thus, interpretation of dielectric relaxation processes at MHz or even kHz frequencies in terms of single molecule rotation is not likely to be correct. The low-frequency relaxations observed in liquid crystals are the result of collective molecular motion, although the models outlined above are useful in analyzing results and comparing materials.

## 3. Discussion

### Dynamic Characterization on Glass Forming

We will now focus on the temperature range below the transition N-N_TB_. Only two longer dimers, with seven carbons in the link (i.e., CBSC7SCB and CBSC7OCB), show typical glass-forming behavior on cooling [40]. We used phenomenological equations and the Vogel–Fulcher–Tammann formula, Equation (2), to describe the temperature dependence of relaxation time data (*τ*). The results of fitting are shown in Figure 1a,b as dashed lines. VFT parameters obtained for Equation (2) are listed in Table 1. It should be stressed, as can be observed in Table 1, that two different dielectric glass transition temperatures are obtained, though rather close to one another, within about 1 K. It is also interesting to note that the pre-factor τ_0_ for the **m**_2_ mode is of the order of 10^−11^ s.

**Figure 2:**
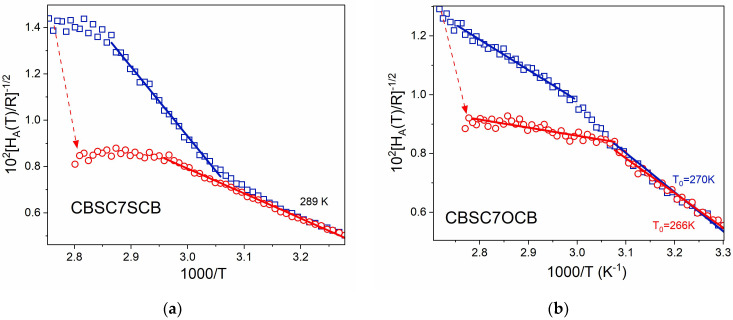
Plots show a linear dependence ${\left[H_{A}\left(T\right)/R\;\right]}^{-1/2}$ on the inverse temperature in N_TB_ phase. (**a**)—CBSC7SCB dimer, (**b**)—CBSC7OCB. The red arrows show the jump of the *H_A_* that omits the critical fluctuation at the N-N_TB_ transition, where *H*_A_ is denoted as the apparent enthalpy of the activation. Symbols: red circles (**○**)—**m**_1_ mode, dark blue squares (**□**)—**m**_2_ mode, solid line: model fitting according to Equation (9). The fitting parameters are listed in Table 2.

A more detailed method to study the dynamics of a glass-forming behavior is to analyze the derivatives of the relaxation time with respect to the inverted temperature [43,44]. Application of this procedure by Rzoska and Drozd-Rzoska leads to the relationship

(9) $${\left[\frac{d\;ln\tau }{d\left(1/T\right)}\;\right]}^{-1/2}={\left[\frac{H_{A}\left(T\right)}{R}\;\right]}^{-1/2}=B^{-1/2}\left(1-T_{0}/T\right),$$

where *H*_A_ is denoted as the apparent enthalpy of activation. In terms of the applicability of the VFT equation,$\;{\left[H_{A}\left(T\right)/R\;\right]}^{-1/2}$ is predicted to have a linear dependence on inverse temperature. The results of applying this equation to both modes are shown in Figure 2a for CBSC7SCB and Figure 2b for CBSC7OCB.

In the case of CBSC7SCB, and mode **m**_2_, the activation enthalpy (almost constant in N phase, H_A_ = 42 kJ/mol) begins to increase in the N_TB_ phase showing a region of linearity (of ${\left[H_{A}\left(T\right)/R\;\right]}^{-1/2}$) down to 325 K (1000/T ≅ 3.08 K^−1^). On the contrary, the activation enthalpy of the mode **m**_1_ jumps at transition N-N_TB_ (from 54 kJ/mol to 113 kJ/mol) then increases, but less rapidly, to join **m**_2_ below 325 K, and from this temperature, both modes have the same enthalpy of activation, probably due to cooperative behavior. Similarly, mode **m**_2_ for CBSC7OCB gradually increases activation enthalpy in the N_TB_ phase (from 53 kJ/mol at transition temperature up to 116 kJ/mol, additionally showing linear region (${\left[H_{A}\left(T\right)/R\;\right]}^{-1/2}$) down to 330 K (1000/T ≅ 3.03 K^−1^)). In this case, mode **m**_2_ shows two linear domains: first from the transition temperature down to 330 K and the second below it. This coincides with the temperature when lateral interaction begins to be important [29].

The activation enthalpy, of the mode **m**_1_, again jumps at transition N-N_TB_ (from 53 kJ/mol to 101 kJ/mol) then grows but less rapidly to join the **m**_2_ mode at about 320 K and since then, both mode have the same activation enthalpy (~116 kJ/mol) due to the cooperative behavior. Continuous lines in both figures are linear fits according to Equation (9). 

We now consider the temperature-derivative procedure to be applied to the critical-like description through Equation (3). A linear dependence with temperature is obtained in the region of validity of the critical-like descriptions according to the relationship

(10) $$T^{2}{\left[\frac{d\;ln\tau }{d\left(1/T\right)}\;\right]}^{-1}={\left[\frac{H_{A}\left(T\right)}{T^{2}R}\;\right]}^{-1}=\Phi^{-1}\left(T-T_{c}\right),$$

**Figure 3:**
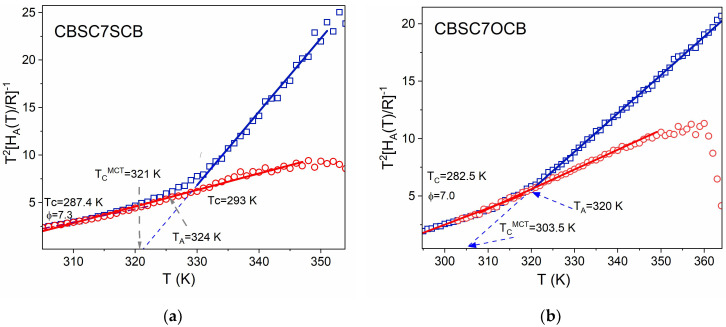
Results of the temperature-derivative analysis (Equation (10)) in N_TB_ phase applied to both **m**_1_ and **m**_2_ modes in which linear dependences indicate domains of validity of the critical-like description; (**a**) symmetric dimer (CBSC7SCB), (**b**) asymmetric dimer (CBSC7OCB). Symbols: red circles (**○**)—**m**_1_ mode, dark blue squares (**□**)—**m**_2_ mode, solid lines: model fitting according to Equation (10). The fitting parameters are listed in Table 3.

The *T_C_* in the above MCT critical-like equation accounts for the crossover temperature from the ergodic to the non-ergodic domain. The critical temperature *Tc* seems to correlate with the caging temperature *T_A_*. Experimental data for both modes are shown in Figure 3 as a plot of [T^2^R/H_A_(T)] vs. temperature. It should be stressed that for both dimers, the data of the **m**_1_ mode exhibit a linear behavior (i.e., follow the DS model) over the entire temperature range of the N_TB_ mesophase. The temperature *T*_C_ is the temperature of the virtual phase transition (the critical temperature), usually located slightly below *T_g_*. The exponent *Φ* ≈ 6–15 was suggested as universal for glass-forming polymers. In contrast, **m**_2_ data in the N_TB_ mesophase clearly show two linear domains, one at low temperatures (DS model) from *T_g_* up to about 320–324 K (denoted as *T_A_* in the figures); for both modes, they seem to be indistinguishable from behavior of the **m**_1_ mode and another domain at high temperatures (MCT-like description) from *T_A_* up to the vicinity of the N_TB_-N phase transition. The crossover temperature coincides well with IR spectroscopy observation for the temperature when lateral interaction increases their strength [29]. Below that temperature, it seems, both modes become coupled and can be described in the same way.

The subsequent linear fittings according to Equation (10) yield the values of the parameters for each mode and dynamic domain, namely, *T*_C_ and the exponent *Φ*. The final fitting of the relaxation data according to Equation (3) is focused on the pre-factor **τ**_0_. All these derived parameters are listed in Table 3 and results of the fittings are drawn in Figure 3.

One of the most noticeable results in Table 3 is related to both the *T*_C_ and *T*_A_ temperatures corresponding to the **m**_1_ and **m**_2_ modes. It seems that a common critical temperature (≈273 K) for the virtual phase transition and also the same glass transition temperature of about 276 K are obtained for both modes. As for the exponent, in the DS domain, *Φ* is 7.0 (**m**_1_ mode) and 7.4 (**m**_2_ mode), typical values usually reported for spin-glass-like systems. In the MCT domain for the m_2_ mode, *Φ* is within the usual range of values.

The application of the temperature-derivative procedure to Equation (4) does not allow for a similar simple linearization methodology as in the previous eq. Instead, an enthalpy function is described with two uncorrelated variables (*K* and *C*) in the form

(11) $$\;\;\left[\frac{d\;ln\tau }{d\left(1/T\right)}\;]=[\frac{H_{A}\left(T\right)}{R}\;\right]=K\left(1+C/T\right)e^{C/T}$$

**Figure 4:**
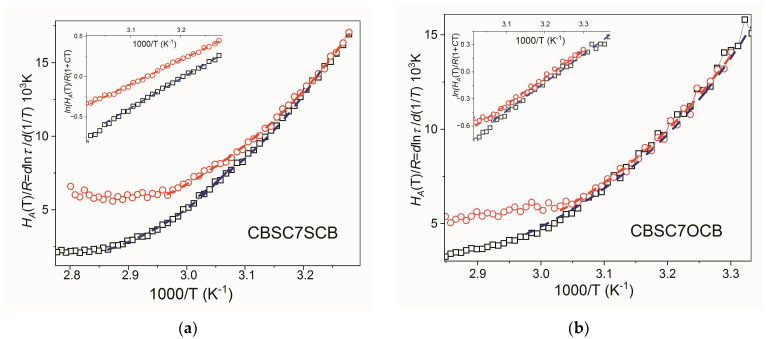
Results of the temperature-derivative analysis (Equation (11)) in N_TB_ phase applied to both **m**_1_ and **m**_2_ modes of (**a**) CBSC7SCB and (**b**) CBSC7OCB. Dashed lines (red and black) indicate dynamic domains of validity of the critical-like description. Symbols: red circles (**○**)—**m**_1_ mode, black squares (**□**)—**m**_2_ mode.

Now, we can obtain *K* and *C* parameters and, consequently, the glass transition temperatures for both relaxation modes. The fitting procedure is unable to fit to only one dynamic domain for each mode as observed in Figure 4, but it allows us to better distinguish the different dynamic domains. It should be stressed that according to Equation (10) and the results of the fits for the *C* and *K* parameters (see Table 4), ln[*H_A_*(*T*)/*R*] exhibits a nearly linear dependence with the inverse of temperature. As suggested recently [44], to proceed further with a linearized expression, Equation (11) can be written as

(12) $$ln\left[\frac{H_{A}\left(T\right)}{R\left(1+C/T\right)}\;\right]=lnK+C/T$$

Different crossover temperatures for each set of relaxation modes, one at about 300 K for m_2_ data and another at about 330 K for m_1_ data, can be observed. The former has not been appreciated for VFT or critical-like analyses of m_2_ data. However, the latter has already been observed in the VFT analysis of m_1_ data and apparently coincides with the estimation of the caging temperature, *T*_A_, shown with the m_2_ data. The final fitting of the relaxation time data according to Equation (4) leads to the pre-factor *τ_0_*. All the (WM)-fitting parameters, *C*, *K*, and τ_0_, for each dynamic domain and relaxation mode are listed in Table 4. The final fits are also drawn in Figure 4. If we focus on the results for *τ*_0_ and *T_g_* (see Table 4), a strong parallelism with the VFT results can be observed. Again, *τ*_0_ for the m_2_ mode is of the order of 10^−10^ s (high-temperature dynamic domain), far from 10^−14^ s. As for the glass transition temperature, two different values are obtained, but less than ca. 3K apart.

## 4. Materials and Methods

**Figure 5:**
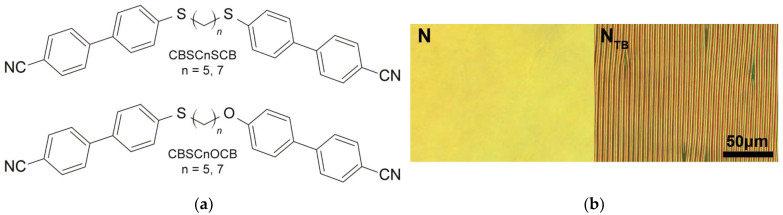
(**a**) Molecular structure of symmetric (CBSCnSCB) and asymmetric (CBSCnOCB) dimers. (**b**) POM textures of the 5 μm planar cell for the CBSC7OCB sample in the N phase (375 K) and the N_TB_ phase (355 K) (cooling rate of 5 °C/min). The scale bar equals 50 μm.

In this study, we investigated both symmetrical and asymmetrical liquid crystal dimers featuring cyanobiphenyl (CB) mesogenic groups. The symmetrical dimers, denoted as CBSCnSCB, incorporate a thioether bridge (S-Cn-S) with varying linker lengths, specifically, either five or seven methylene groups (n = 5, 7). On the other hand, the asymmetrical dimers, referred to as CBSCnOCB (n = 5, 7), connect the mesogens to an alkyl chain with either five or seven methylene groups on one side via a thioether bridge and on the other side via an ether bridge. The molecular structures and typical textures in the nematic and twist-bend nematic phases for investigated compounds are illustrated in Figure 5, and for comprehensive details regarding the synthesis of thioether/ether dimers, we refer to the cited references [45,46].

**Figure 6:**
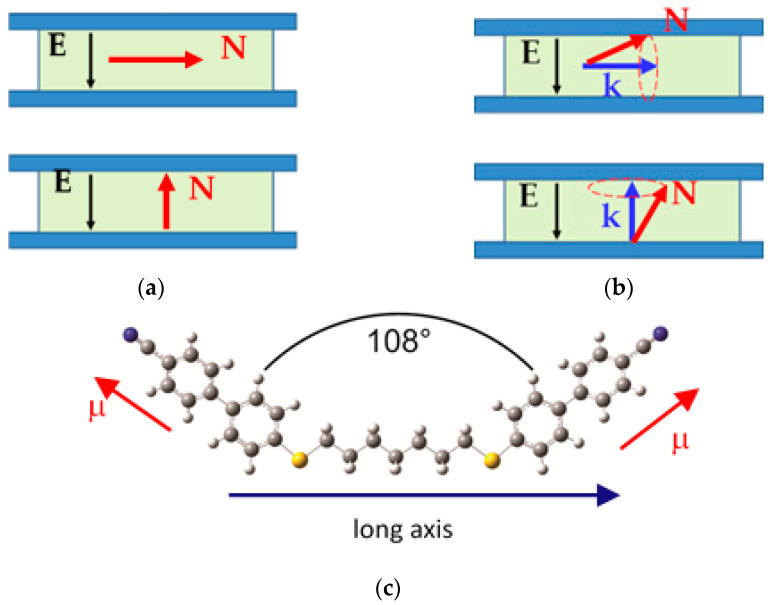
Laboratory and molecular frame of reference. Planar (top) and homeotropic cell (bottom) (**a**) in the N phase, (**b**) in the N_TB_ phase, where k is the helical axis, (**c**) molecular frame of reference.

Dielectric spectroscopy investigations were conducted over a broad frequency range spanning from 10 Hz to 1 GHz. These measurements were performed using two impedance/network analyzers, specifically the HP4I92A and HP4I95A, and encompassed samples aligned in both planar and homeotropic orientations. Diverse cell configurations with gap sizes ranging from 1.6 to 12 μm were employed. Homeotropic cells were achieved by using a commercial solution of the AL 60,702 polymer (JSR Korea, Gongju-City, Republic of Korea) and planar cells equipped with SE-130 polymer alignment layers from Nissan Chemical Industries, Ltd. For high-frequency dielectric measurements, we utilized gold-plated cells. Quality of alignment was tested using the ITO cells of the same geometry and alignment agents. Geometry of planar and homeotropic sandwich cells and molecular frame of reference are shown in Figure 6.

For better deconvolution of the modes in the dielectric spectra, we used permittivity derivatives vs. ln(f) [20,24].

**Figure 7:**
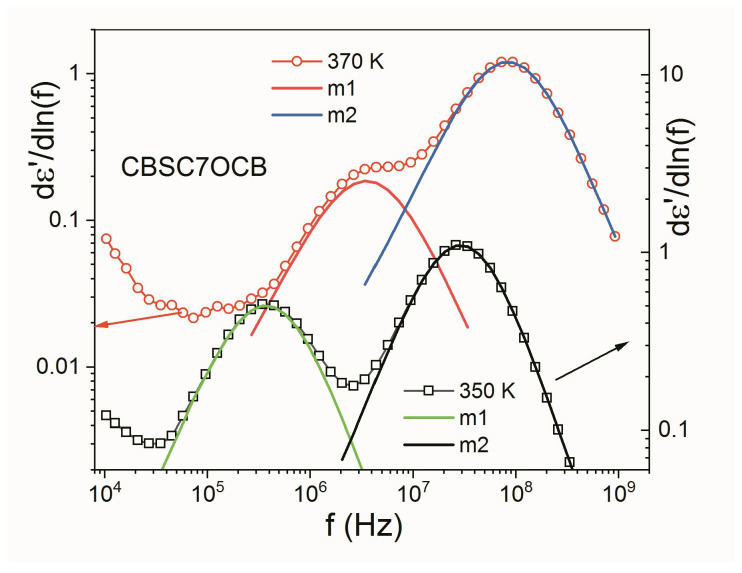
Fitting of the derivative of permittivity vs. ln(f) showing the deconvolution of the molecular modes: **m**_1_ and **m**_2_.

(13) dε’d(lnf)=dε’d(lnω)=∑j=1nReδεjα(iωτj)α[1+(iωτj)α]2,

Here, *δε_j_*, *τ_j_*, *α_j_* are the fitting parameters of the equation of the derivative of *ε*′. Figure 7 shows the fitting examples of the *dε*′/*d*(ln*f*) at two temperatures (370 K, 350 K) and how they reproduce the experimental curve. The errors are less than 5% for frequency and 7% for strength of the mode. The shape parameters were found to be quite small (*α* < 0.08).

## 5. Conclusions

The dielectric relaxation spectra for symmetric CBSC7SCB and asymmetric CBSC7OCB dimers are resolved in terms of two relaxation processes, m_1_ and m_2_, as predicted with the rotational diffusion model. The high-frequency relaxation process originates from the precession rotation of the longitudinal component of the monomer dipole moment of each thiocyanobiphenyl (SCB) dipole moment around the director. The relaxation rate is accelerated with respect to the isotropic relaxation because $\tau_{10}$ is shorter as compared to $\tau_{D}$. The low-frequency relaxation corresponds from the “end-over-end” rotation of the monomer dipole moment. The relaxation rate of that mode is retarded with respect to the isotropic one. It is clear that dynamics of the two relaxation process modes m_1_ and m_2_ are quite different in the two temperature regions of the N_TB_ mesophase. In the first region, which is extended almost 30 K from N-N_TB_ transition, for mode m_1_ assigned to “end-over-end” rotation, activation enthalpy initially jumps almost twice on entering N_TB_ then increases continuously over almost 30 K. This turns out to be well associated with the appearance of longitudinal dipole correlations discovered in IR studies. In the same region, the activation enthalpy of mode m_2_ (assigned to precession of dipoles) grows continuously but much faster than m_1_ and finally they meet at the same value of H_A_(T). This meeting temperature corresponds well to the appearance of transverse interactions as seen in IR measurements. The description of the critical behavior of dynamic domains related to cooperative molecular motions is quite sufficient to adequately describe the dynamics associated with the two main molecular motions in the N_TB_ mesophase of the symmetric and asymmetric dimer. Both types of molecular motion appear to strongly cooperate at low temperatures, changing in a coordinated manner as the temperature of the material approaches the glass transition point. As expected, it turns out that both molecular motions determine glassy dynamics with the same glass transition temperature *T_g_*.

## Figures and Tables

**Table 1:** Fitting parameters according to Equation (2) for the different dimers and the calculated glass transition temperature for the **m**_1_ and **m**_2_ modes.

Sample	Mode	log_10_ [τ(s)]	T_0_ (K)	B (K)	T_gl_ (K)
CBSC7SCB	**m_1_**	−10.1	266.6	682.6	289.8
	**m_2_**	−11.9	265.6	631.9	286.3
CBSC7OCB	**m_1_**	−9.2	261.3	596.0	284.3
	**m_2_**	−11.5	260.5	621.7	280.5

**Table 2:** Fitting parameters according to Equations (2) and (9) for the different dimers.

Sample	log_10_ [τ(s)]	Equation (2)	Equation (9)	Range [1000/T(K^−1^)]
CBSC7SCB	−10.3	269	289	2.96–3.3
	−11.6	264	289	3.15–3.3
CBSC7OCB	−8.9	265	266	3.08–3.3
	−11.5	261	270	3.08–3.3

**Table 3:** Fitting parameters according to Equation (10) for the different dimers.

Sample	T_A_	Φ (T < T_A_)	Tc	Φ	Range (K)	Description
CBSC7SCB	324 K	287.4 7.3	293	7.3	305–347	DS
			321	1.3	330–351	MCT
CBSC7OCB	320 K	282.5 7.0	287.4	7.0	295–350	DS
			303.5	1.3	320–365	MCT

**Table 4:** Fitting parameters according to Equation (11) for the different dimers.

Sample	log_10_ [τ(s)]	K (K)	C (K)	T_gl_ (K)	Range (1000/T (K^−1^))
CBSC7SCB	−7.78	0.0789	3016	268.2	2.97–3.28
	−9.08	0.0082	3653	267.9	3.02–3.22
CBSC7OCB	−7.15	0.058	3028	263.9	3.03–3.30
	−9.05	0.031	3198	260.6	2.98–3.35

## Data Availability

ZENODO will be the main platform for data storage after the project will be finished. https://zenodo.org/ (accessed on 1 January 2024).
